# Single Cell and Spatial Transcriptomics Identify Novel Immune-Stromal Interactions in Cardiac Allograft Vasculopathy

**DOI:** 10.21203/rs.3.rs-7812112/v1

**Published:** 2025-11-21

**Authors:** Macee C Owen, Daniel Yuhang Li, Haewon Shin, Wenduo Gu, Alekhya Parvathaneni, Farid F Kadyrov, Xiaoran Wang, Maura Sticco-Ivins, Gianni Bonnici, Samantha L. Nelson, Hao Dun, Sariah Hyacinth, Michael T. Cain, Albert Pedroza, Alex Dalal, Karim Sallam, Jack Boyd, Joseph Woo, Junedh M Amrute, Paul Cheng, Kory J Lavine, Benjamin J Kopecky

**Affiliations:** 1 Division of Cardiology, Department of Medicine, Washington University School of Medicine, St. Louis, MO.; 2 Division of Cardiovascular Medicine, Stanford University School of Medicine, Palo Alto CA.; 3 Center for Systems Biology, Massachusetts General Hospital and Harvard Medical School, Boston, MA.; 4 Department of Radiology, Massachusetts General Hospital and Harvard Medical School, Boston, MA.; 5 Division of Molecular Microbiology, Washington University School of Medicine, St. Louis, MO.; 6 Department of Medicine, Division of Cardiology, University of Colorado Anschutz Medical Campus, Aurora, CO.; 7 Department of Surgery, Division of Cardiothoracic Surgery, University of Colorado Anschutz Medical Campus, Aurora, CO.; 8 Department of Cardiothoracic Surgery, Stanford University School of Medicine, Palo Alto, CA.

## Abstract

Cardiac allograft vasculopathy (CAV) is the leading cause of mortality in heart transplant recipients. Despite the prevalence of CAV, there are no targeted therapeutic options to prevent or reverse disease progression, and patients ultimately require retransplant. CAV is defined by progressive neointimal hyperplasia in donor heart coronary arteries, leading to luminal obliteration and ultimately allograft failure or sudden cardiac death. Although immune and stromal cell interactions are believed to play a key role in CAV pathogenesis, the specific cellular players and molecular signals driving disease remain undefined. In this study, we leverage single-cell RNA sequencing and spatial transcriptomics of human coronary arteries to transcriptionally characterize CAV and define the neointimal microenvironment. We compare arteries with CAV to atherosclerotic coronary artery disease and non-disease controls to identify a unique CAV transcriptional signature. Integration of single-cell RNA sequencing and spatial transcriptomic datasets revealed that modulated vascular smooth muscle cells and macrophage subsets dominate the CAV neointima and suggest that these cells interact to propagate type 1 interferon (IFN)-mediated inflammation. In a mouse CAV model, we demonstrate that interferon blockade with Ruxolitinib significantly reduced the incidence of CAV and prolonged allograft survival. Collectively, this study offers a novel and detailed characterization of the unique cellular and transcriptional landscape of CAV and identify a candidate pathway that may underly CAV pathogenesis, which could serve as a new therapeutic target for this devastating disease.

## Introduction

Heart transplantation is a lifesaving procedure for patients with congenital heart diseases and end-stage heart failure. However, prevailing strategies to prevent rejection are imperfect and both acute and chronic allograft rejection remain common. While short-term outcomes have improved since the advent of heart transplantation due to advances in immunosuppression and surgical technique^1,2^, long-term survival remains relatively unchanged^3^ as patients face an elevated lifelong risk of developing malignancies, infection and chronic rejection^4^. A form of chronic allograft rejection termed cardiac allograft vasculopathy (CAV) is the leading cause of mortality in heart transplant recipients, affecting nearly 50% of recipients within 10 years of transplant^5^.

CAV is characterized by a progressive, concentric thickening of the neointima of donor coronary arteries^6,7^. Over time, the formation of this neointima in vasculature supplying the heart causes luminal occlusion, resulting in tissue hypoxia and allograft failure. The clinical presentation of CAV varies, ranging from symptoms of heart failure in some patients to severe arrhythmias or even sudden cardiac death in others^8,9^. Fortunately, the use of advanced diagnostic modalities has improved our ability to identify CAV at its early stages. Intravascular ultrasound (IVUS) performed concurrently with coronary angiography is the primary diagnostic tool for detecting CAV^10^. However, the need for frequent IVUS procedures as routine transplant follow-up care is both invasive and costly. Current strategies to slow CAV progression are focused on prevention of neointimal development and include Mammalian Target of Rapamycin (mTOR) inhibitors and statins^9,11–14^. However, these therapies are insufficient as patients with severe CAV ultimately require repeat heart transplant. Given the scarcity of available donor organs and the increased risk for suboptimal outcomes with retransplant^9^, there is a significant need for new therapies for CAV.

While the pathogenesis of CAV remains poorly understood, the prevailing opinion is that there are unique risk factors and mechanisms compared to atherosclerotic coronary artery disease (CAD)^15,16^. Previous studies have focused on the contribution of endothelial dysfunction and T cell immunity in CAV^17–22^, with few studies investigating the role of macrophages^23,24^. Others have demonstrated that smooth muscle cells (SMC) undergo phenotype-switching and proliferate to form the neointima^25,26^. SMC phenotypic modulation coupled with immune activation are common features across many different etiologies of vascular diseases. The precise mechanisms by which immune and stromal cells interact in CAV remain unclear. The advent of high-throughput, high-resolution sequencing technologies, alongside expansion of human tissue biobanks, provides an unparalleled opportunity to elucidate the transcriptional and spatial landscape of CAV. In this study, we performed scRNA-seq and spatial transcriptomics of coronary arteries from explanted human hearts obtained from CAV patients at the time of heart re-transplantation and compared them to CAD and non-diseased arteries in explanted hearts. To our knowledge, only one other study has investigated CAV using earlier versions of spatial transcriptomic technology^26^. By leveraging scRNA-seq and current spatial transcriptomics, we uncovered cell states that emerge during CAV pathogenesis, mapped these cells to their spatial neighborhoods, and identified putative signaling pathways involved in cell-cell communication within these disease niches. Our findings suggest that allograft-infiltrating macrophages are central to sustaining the chronic inflammatory state where they propagate type I IFN signaling, express proteases that may contribute to elastin fragmentation, and drive neointimal proliferation. Spatially, inflammatory macrophages are distributed throughout the neointima and interact with modulated SMCs via TGFβ, IL-1 family and type 1 IFN signaling pathways. In a mouse model of CAV, we assessed the role of type 1 IFN and found that inhibition of these signaling pathways improved transplantation outcomes and drastically reduced the incidence of vasculopathy. Our findings point to multifaceted roles of myeloid and stromal cells in CAV progression, laying the groundwork to explore new therapeutic targets for CAV.

## Methods

### Human coronary artery tissue collection (scRNA-seq)

Fresh human coronary arteries were obtained through the Stanford CT surgery biobank (CAD/CAV samples) as well as a collaborative effort with Donor Network West (control arteries), following a protocol approved by both Donor Network Wesťs research committee and Stanford University's Institutional Review Board (IRB #32769, #54213). Explanted hearts used for scRNA-seq were obtained as part of the Chan Zuckerberg Initiative funded Human Arterial Atlas (Zhao Q 2024). For this study, we collected coronary arteries from both CAV and CAD explanted hearts while control samples were collected from deceased donors whose hearts did not have an active clinical match and thus were donated to research. Given the scarcity of this resource, the primary consideration during collection was to ensure the integrity and viability of tissue for downstream analysis. Explanted coronaries from control or explanted hearts were collected without performing additional skin incisions or superficial dissections, adhering to research ethics guidelines and showing respect for the organ donors. This approach ensured that our research activities did not interfere with the donor's appearance or the transplantation process. Upon collection, fresh coronary arteries were immediately placed in cold Hank's buffered saline solution (HBSS, Sigma 14025076) to maintain tissue viability during transportation and processing. For consistency, we always targeted middle segments (>1cm away from any bifurcation) of the coronary vessel for collection.

### Human coronary artery digestion (scRNA-seq)

Immediately after collection, fresh coronary artery tissues were separated into equal 50mg pieces. These samples were then placed in a digestion solution comprising Liberase TM (10.4U/mL working concentration in HBSS, Sigma-Aldrich 05401127001) and elastase (8U/mL, Sigma E7885 in HBSS) and were then finely minced on ice using iris scissors. The resulting digested product was transferred to a 2.0ml Eppendorf tube and subjected to constant agitation at 650RPM in a heated Eppendorf Thermomixer (Model 5382). To enhance heat exchange, distilled water was added to the heating block. The digestion mixture was monitored at 30 and 45 minutes under the microscope to assess for single cell suspension, with vigorous pipetting (3X) performed if undigested cell clumps were observed. After 60 minutes of digestion at 37°C, the digested products were vigorously pipetted 10 times and then filtered through a 70μm strainer using wide-bore pipette tips. The filtered solution was centrifuged at 500g for 10 minutes in V-bottom plates to optimize cell collection. The resulting cell pellet was resuspended in fresh, cold HBSS. Finally, the samples underwent FACS (Fluorescence-Activated Cell Sorting) using Calcein green (ThermoFisher C34852) to assist in live/dead discrimination. The sorted cells were used immediately for single-cell capture and subsequent library preparation.

### Single-cell capture and sequencing (scRNA-seq)

Single-cell captures and library preparation were performed using chemistry version 3 (V3) according to 10x Genomics instructions. Cells were loaded into 10x Genomics microfluidics chip G for encapsulation with their proprietary barcoded gel beads in the 10x Genomics Chromium X controller. Single-cell libraries were constructed according to manufacturer’s instructions with index labeling using the Dual Index Kit TT set A. This allows for library pooling into one lane for sequencing on the Illumina NovaSeq6000 platform with a targeted read depth of 75,000 reads per cell.

### Data alignment, quality control and cell type annotation (scRNA-seq)

Raw FASTQ files were aligned to the GRCh38 reference genome (v) using Cell Ranger (10x Genomics, v6.1). Subsequent quality control (QC), normalization, dimensional reduction, and clustering was performed with Seurat v4.0. Cells were filtered using the following thresholds: 500 < nFeature_RNA < 7000; nCount_RNA < 30,000; percent mitochondrial reads < 10. Raw RNA counts from cells that met these criteria were normalized and scaled using SCTransform regressing out percent mitochondrial reads and nCount_RNA. Principal component analysis (PCA) was performed on the normalized RNA counts, and the number of PCs used for downstream clustering was dependent on the following criteria: PCs cumulatively explain > 99% of the variation present in the data and percent variation associated with the PCs is less than 1%. Weighted nearest neighbor clustering (WNN) was performed with the significant RNA PCs directly without PCA as previously outlined with the FindMultiModalNeighbors function in Seurat. Subsequently, a uniform manifold approximation (UMAP) embedding was constructed and FindClusters was used to cluster cells using the Smart Local Moving modularity optimization algorithm in an unbiased manner. Clustering was performed for a suite of different resolutions (0.1–0.8 at 0.1 intervals) and differential gene expression testing was done using the FindAllMarkers function and a Wilcoxon Rank Sum test with a logFC cut-off of 0.25 and a min.pct cut-off of 0.1. Clusters were annotated using canonical gene markers and subsequent dot plots (RNA) were created to assess clean separation of clusters into distinct cell types. Previously identified canonical marker genes were also plotted on the UMAP object to further validate cluster annotations.

### Differential Gene Expression (scRNA-seq)

We use the DESeq2 package to perform DE analysis. After QC, cells were subsetted for each cell population annotated from the global reference, raw counts were aggregated to the patient level, data normalized using a regularized log transform, and differential expression analysis between control and diseased samples via DESeq2. Genes were deemed statistically significant if adjusted p-value < 0.05 and absolute(log2FC) > 0.5.

### Pathway Analysis (scRNA-seq)

Statistically significant DE genes were used to perform pathway analysis via EnrichR (https://maayanlab.cloud/Enrichr/). Pathway enrichment values were downloaded as .txt files and plots generated with EnrichPlot and EnrichR packages in Seurat.

### Myeloid, smooth muscle, fibroblast, and endothelial analysis (scRNA-seq)

To cluster cell types into distinct cell states, we subsetted the cell type of interest, re-normalized, computed PCAs, harmony integrated, computed UMAPs, and clustered data at a range of resolutions. DE analysis was then used to identify marker genes for each cell state and a dot-plot to assess clustering separation. Using the top marker genes we calculated gene set z-scores and plotted them in UMAP space.

### Human coronary artery collection (Spatial Transcriptomics)

Acquisition of coronary artery samples for spatial transcriptomics was approved by the Washington University institutional review board (IRB 201104172). Coronary arteries were collected at time of cardiac explant and were fixed in PFA and embedded in paraffin blocks. Blocks were stored at 4C, covered from light.

### Human coronary artery collection

Additional coronary arteries were obtained per approved IRB #01568 at the University of Colorado Anschutz Medical Campus. Coronary arteries were collected at time of cardiac explant and were fixed in PFA and embedded in paraffin blocks. Blocks were stored at 4°C, covered from light.

### Spatial transcriptomics sample preparation

DV200 (percentage of total RNA fragments >200 nucleotides) values for each tissue were calculated and tissues with DV200 values of <15% were excluded from downstream sample preparation. Visium CytAssist Spatial Gene Expression for FFPE protocol was used to prepare Hematoxylin and Eosin (H&E) stained tissue sections for use with Genomics Visium CytAssist Spatial Gene Expression for FFPE assay. In brief, paraffin embedded coronaries were hydrated in ice bath prior to sectioning. 5 um slices were sectioned with a clean blade on a microtome and sections were placed on charged microscope slides. Slides were baked for 3 hours at 42°C and were placed in desiccant beads overnight prior to deparaffinization and staining. Tissues were passed through multiple exchanges of xylenes/ethanol for dehydration/rehydration prior to staining with H&E per Visium Spatial Gene Expression for FFPE tissue staining instructions. Capture regions were demarcated, and high-resolution images were obtained with the Zeiss Axioscan Z1 (Washington University Center for Cellular Imaging) prior to submission for barcoding, sequencing and spatial alignment via Space Ranger at The McDonnell Genome Institute.

### Integration of spatial transcriptomics data

Visium 10x data from 10x Genomics was processed similarly to the scRNA-seq data with Seurat v4. Data was re-normalized using SCTransform to keep analysis consistent with the scRNA-seq data processing. The scRNA-seq reference map was used to impute voxel annotations from selected spatial transcriptomic samples; additionally, mapping scores and gene signatures were plotted to validate the mapping. Seurat deconvolution and SPOTlight (v0.1.7) were used for spot deconvolution and calculating Pearson correlation coefficients to identify cells that co-localized in space.

### Reference mapping of scRNA-seq and spatial transcriptomic data

Briefly, the scRNA-seq data was normalized using SCTransform while the annotated spatial transcriptomic data was used as the reference. FindTransferAnchors was used with a PCA reference reduction and 40 components. These anchors were then passed into the MapQuery function to impute cell annotations and project the scRNA-seq data onto the spatial transcriptomic UMAP embedding.

### Receptor-ligand interaction analysis

For comprehensive cell-cell communication analysis, we first used the MultiNicheNetr (v2.0.1) default pipeline to explore the ligand-receptor interactions from myeloid cells, T cells, endothelial cells, B cells, fibroblasts and SMCs in the scRNA-seq data. Bubble plots were generated to represent the top 50 interactions between cells.

To explore specific ligand-receptor interactions in select cells of interest, we next used the NicheNetr (v2.1.5) default pipeline, examining interactions between T cells, macrophage, and SMC subpopulations. The regulatory potential from the top 20 ligands on their predicted targets was represented in a heatmap.

### Histology and Immunofluorescence

Paraffin-embedded coronary artery samples were fixed for 24 hours at 4°C in 4% paraformaldehyde, washed in 1X PBS, and embedded in paraffin. Paraffin-embedded sections were cut at 5 um thickness using a microtome. Slides were baked at 60 °C for 1 hour, deparaffinized with serial xylene washes, and rehydrated with ethanol. Slides underwent methanol treatment (10% MeOH + 3% H_2_O_2_) for 20 min at RT followed by 3X TBS-T washes (5 min each). Antigen retrieval was performed using the AR6 buffer (Akoya Biosciences, AR600250ML) for 15 min in the microwave and then cooled to room temperature. Tissue sections were marked with a hydrophobic pen and blocked in 10% BSA in TBS-T for 30 minutes at room temperature. Slides were then stained with the primary antibody diluted in 10% BSA in TBS-T overnight at 4 °C (α-Smooth Muscle Actin (1A4) (Cell Signaling Technology #46469S); CD68 (BioRad clone KP1); CD4 (Abcam clone EPR6855); PNAd (BioLegend clone MECA-79); Phospho-Stat1 (Cell Signaling Technology #9167); TNFRSF11B (Abcam clone ab73400)). Next, the primary antibody was detected using Opal Polymer HRP Ms + Rb (PerkinElmer Opal Multicolor IHC system). The PerkinElmer Opal Multicolor IHC system was utilized to visualize antibody staining per manufacturer protocol. Slides were imaged using a Zeiss Axioscan Z1 automated slide scanner. Image processing was performed using Zen Blue and Zen Black (Zeiss). Verhoeff-Van Gieson (VVG) staining was performed using an Elastic, Verhoeff Stain Kit (Newcomer Supply) per manufacturer’s instructions. H&E staining was performed using Mayer’s Hematoxylin (Dako, Lillie’s Modification lot 11574498), Bluing buffer (Dako, lot 177177), and Eosin Y Solution Alcoholic (Sigma-Aldrich, lot SLCQ5239) as part of spatial transcriptomics preparation per instructions in Visium Spatial Transcriptomics for FFPE protocol.

### Multispectral IHC- PhenoImager HT 9-color

Through our collaboration with the Human Immune Monitoring Shared Resource (HIMSR) at the University of Colorado School of Medicine, we performed multispectral imaging using the PhenoImager HT instrument (formerly Vectra Polaris, Akoya Biosciences). Briefly, the slides were deparaffinized, heat treated in antigen retrieval buffer, blocked, and incubated with primary antibodies [SMA (DAKO cat #m0851 1:500); CD31 (abcam cat#182981 1:100)], followed by horseradish peroxidase (HRP)-conjugated secondary antibody polymer, and HRP-reactive OPAL fluorescent reagents. The slides were stripped between each stain with heat treatment in antigen retrieval buffer. Whole slide scans were collected with PhenoImager HT v2.0.0 software using the 20x objective with a 0.5 um resolution. Regions of interest were selected and rescanned using the 20x objective and the multispectral imaging cube. Spectral references and unstained control images were measured and inForm software v3.0 was used to create a multispectral library reference. The multispectral images (.im3 files) were spectrally unmixed analyzed using inForm software v3.0.

### Animal Models

Mice were purchased from Jackson Laboratories (Bar Harbor, MD, USA) and housed at the University of Colorado Anschutz Medical Campus, Animal Facility under standard conditions. All animal procedures were approved by the Institutional Animal Care and Use Committee (IACUC) #01386 at the University of Colorado Anschutz Medical Campus. Mouse strains used included C57BL/6J (Jax #000664) and B6 (C)- H2-Ab1bm12/KhEgJ (Jax #001162). All donor and recipient mice were age- and sex-matched between 8 and 12 weeks of age. Ruxolitinib (Biotechne/Tocris #7064) was incorporated into Teklad 2920x (Research Diets #C25022701i) at 2g Ruxolitinib per kg chow. Immediately after surgery, recipient mice were fed standard chow. After seven days, mice were either continued on standard chow or placed on Rux-chow for the duration of the study. Humane endpoints were continuously monitored to ensure tolerability and safety of intervention.

### Heterotopic Heart Transplantation

Animals were administered subcutaneous buprenorphine extended-release formulation (1 mg/kg) followed by intraperitoneal ketamine HCl (80 mg/kg) and xylazine (8 mg/kg) dissolved in saline. All mice were continuously monitored during anesthesia. Heart grafts were harvested from donor mice and transplanted heterotopically into the abdomen of recipients after 1 hour of cold (4°C) ischemia, as previously described (Corry, 1973). After transplantation, mice allografts were palpated daily. Cessation of a palpable heartbeat, confirmed by visual inspection, indicated rejection of the cardiac allograft. Perioperative graft loss (within 72 hours) was excluded from the analysis.

### Independent data access and analysis

We attest that authors MCO and BJK had full access to all the data in the study and take responsibility for its integrity and the data analysis.

### Materials and data availability

The data that support the findings of this study are available from the corresponding author on reasonable request.

### Code Availability

All scripts used for scRNA-seq and spatial transcriptomics data analyses are available from GitHub.

## Results

### Myeloid cells are enriched in CAV and drive differential gene expression

**Figure 1.**
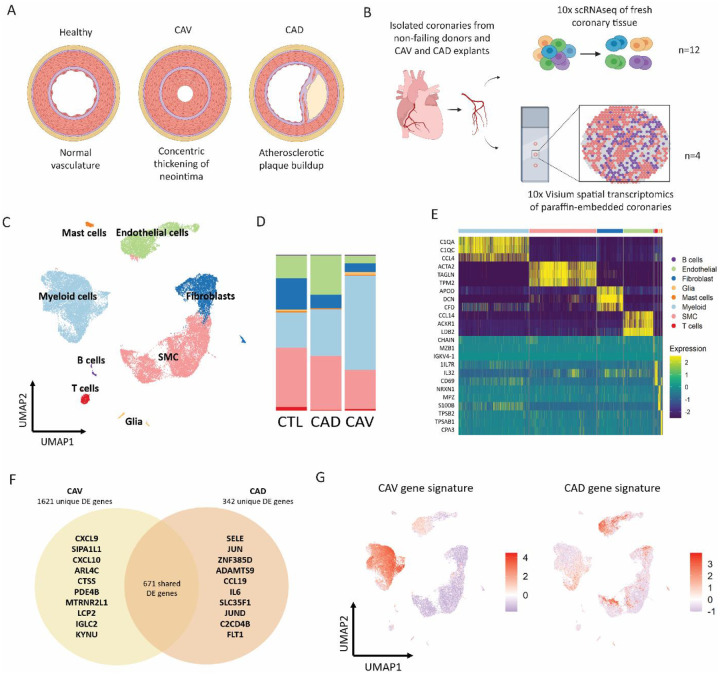
**A** Graphic denoting pathology of experimental disease states (CAV and CAD) compared to a control coronary artery. **B** Schematic of single cell RNA sequencing and spatial transcriptomic workflow. **C** Uniform Manifold Approximation and Projection for Dimension Reduction (UMAP) embedding plot of CAV, CAD and control single cell libraries. **D** Composition plot of cell clusters split by disease state. **E** Heat map of top 5 differentially expressed genes across cell types using statistically significant genes (adjusted P value <0.05, Bonferroni correction). **F** Top unique and shared statistically significant (avg. logFC >0.5, adjusted P value <0.05, Bonferroni correction) DE genes in CAD and CAV samples. **G** Feature plot of CAV and CAD module scores generated from top 50 statistically significant DE genes relative to control.

To characterize the transcriptional landscape and identify disease-specific drivers of CAV, we performed scRNA-seq of fresh explanted human coronaries from CAV patients (4 samples), CAD patients (2 samples), and non-disease control samples (6 samples) isolated in identical manners (Fig. 1A–B). Coronary arteries were included from both male and female hearts. After pre-processing and QC of sequenced samples, we obtained high-quality RNA expression in 31,825 total cells (**Supplemental Fig. 1A**). We then performed dimensional reduction, uniform manifold approximation and projection (UMAP) construction and cell clustering with differential gene expression (DGE) to annotate cell types (Fig. 1C). We identified 8 transcriptionally distinct cell types distinguished by canonical gene markers (Fig. 1C, E, **Supplemental Fig. 1B**). We constructed cell-type-specific gene set scores and detected strong enrichment across clusters (**Supplemental Fig. 1C)**. We observed marked differences in cell type composition across conditions, with a significant expansion of myeloid cells in CAV samples (Fig. 1D). To assess cell-specific transcriptional changes in CAV and CAD, we performed DGE analysis comparing CAV and CAD samples to control samples and observed distinct global gene expression signatures between CAV and CAD (Fig. 1F). Of note, there were 1621 differentially expressed genes (DEGs) unique to CAV, with the top ten upregulated DEGs including *CXCL9, CXCL10, CTSS, PDE4B*, and *IGLC2*. Several of these genes are induced by IFN signaling. Feature plots of type 1 and type 2 IFN genes revealed that type 1 IFN associated genes (e.g., *IFNB1*, *IFNA5*) were predominantly expressed in myeloid cells. We observed no differences in type 2 IFN (*IFNG*) expression in T cells (**Supplemental Fig. 1D**). Other top genes unique to CAV included *CTSS*, which was highly expressed in myeloid cells (**Supplemental Fig. 1E**). To identify which cell types drive differences in gene expression between CAV and CAD groups, we constructed and plotted a gene set score with the top 50 DEGs for CAV and CAD in UMAP space. The gene signature of CAV was localized to the myeloid population (Fig. 1G), suggesting that myeloid cells drive differential gene expression in CAV. This finding is distinct from CAD, where the top DEGs in CAD samples displayed the greatest enrichment in endothelial and stromal cell clusters.

### Type 1 interferon signaling is upregulated in myeloid cells in CAV

**Figure 2.**
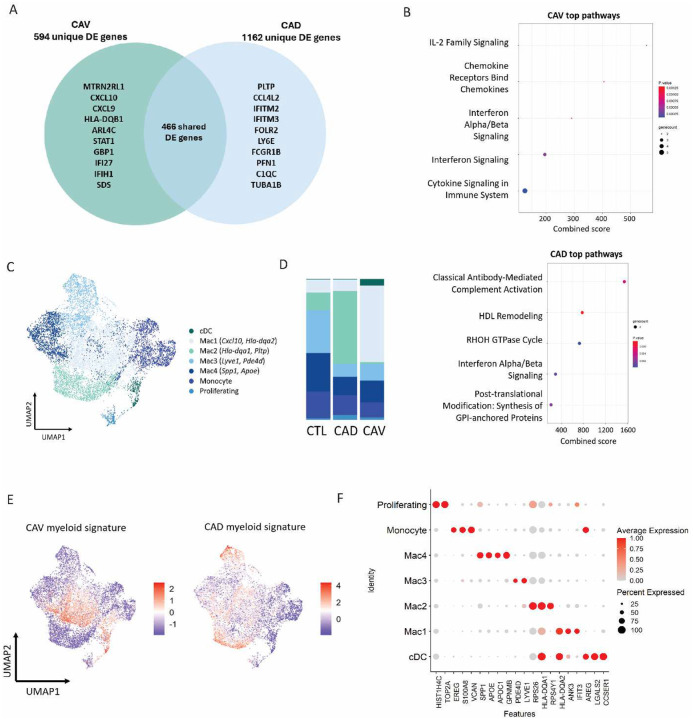
**A** Top unique and shared statistically significant (avg. logFC >0.5, adjusted P value <0.05, Bonferroni correction) DE genes in myeloid cells in CAD and CAV relative to control samples. **B** Pathway analysis of top statistically significant DE genes in myeloid cells in CAV and CAD. **C** UMAP embedding plot of subclustered myeloid cell states and **D** the respective composition plot. **E** Feature plot of CAV and CAD myeloid module scores generated from top 25 statistically significant DE genes relative to control samples. **F** Dot plot of top DE gene expression in myeloid cell states.

To further investigate myeloid cells which were significantly enriched in CAV samples, DGE analysis was performed on myeloid cells in diseased samples (CAV and CAD) relative to control, which revealed 594 DEGs unique to CAV, 1162 DEGs genes unique to CAD, and 466 shared DEGs (Fig. 2A). The majority of highly upregulated DEGs in myeloid cells from CAV samples were associated with type 1 IFN signaling (*CXCL9, CXCL10, STAT1, IFI27, HLA-DQB1*, and *IFIH1*). Pathway analysis of the top 25 DEGs revealed distinctions between CAV and CAD. Top pathways in CAV included pro-inflammatory cytokine and chemokine signaling, including IFN signaling (Fig. 2B). Conversely, top pathways in CAD included HDL remodeling and classical complement activation (Fig. 2B).

To further investigate shifts in myeloid cell states in CAV, we performed subclustering of the myeloid compartment and identified 7 distinct clusters of macrophages, monocytes and classical dendritic cells with unique gene signatures (Fig. 2C, F). Cell composition analysis between conditions revealed a significant expansion of Mac1 (*CXCL10, IFIT2, HLA-DRA)* and cDC (*FLT3, XCR1*) populations in CAV (Fig. 2D). To identify which cell states drive DGE in CAV and CAD, we generated module scores for the top 25 DEGs in myeloid cells and mapped the scores to the subclustered UMAP. In CAV, DEGs were enriched in Mac1 and cDC clusters, while DEGs in CAD predominantly mapped to Mac2 and Mac3 (Fig. 2E). In addition to IFN and classic inflammatory cytokines (*IL-1*β*, TNF*), other top genes expressed in Mac1 included *CXCL8, CCL3* and *CCL4*. Collectively, these results suggest that the Mac1 cell state is expanded in CAV and that this population of cells may be involved in disease pathogenesis.

### Inflammatory modulated smooth muscle cells are enriched in CAV

**Figure 3.**
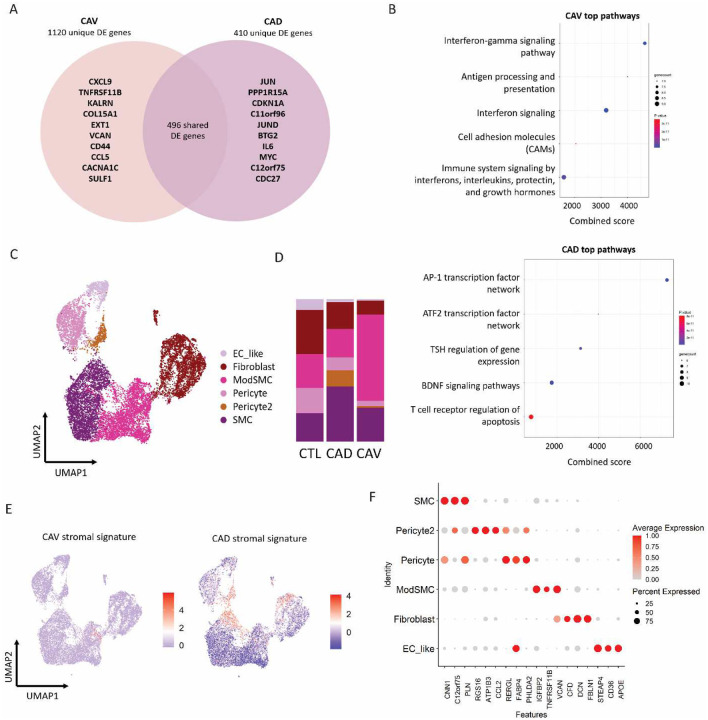
**A** Top unique and shared statistically significant (avg. logFC >0.5, adjusted P value <0.05, Bonferroni correction) DE genes in stromal cells in CAD and CAV relative to control samples. **B** Pathway analysis of top DE genes in stromal cells in CAV and CAD. **C** UMAP embedding plot of subclustered stromal cell states and **D** the respective composition plot. **E** Feature plot of CAV and CAD stromal module scores generated from top 25 statistically significant DE genes relative to control. **F** Dot plot of top DE gene expression in stromal cell states.

Given the widely appreciated role for stromal cells in CAV and CAD pathobiology, we performed DGE analysis on stromal cells and identified 1120 DGEs unique to CAV, 410 DEGs unique to CAD, and 496 shared DGEs (Fig. 3A). Among the top 10 DGEs in stromal cells in CAV we identified *CXCL9*, *VCAN*, *CCL5*, *COL15A1* and *CD44*. We performed pathway analysis using the top 25 CAV and CAD DEGs (Fig. 3B). CAV stromal cells were enriched for IFN signaling, antigen presentation, cell adhesion and immune system signaling (Fig. 3B) whereas CAD stromal cells expressed genes associated with pathways related to AP-1 transcription, ATF2 transcription, and TSH signaling.

We subclustered stromal cells and identified 6 distinct smooth muscle cell (SMC), fibroblast and pericyte cell states (Fig. 3C, F). Cell composition analysis revealed marked expansion of modulated SMCs (*FN1, TNFRSF11B, COL8A1, CCN2*) in CAV (Fig. 3D). We generated gene signature module scores with the top 25 DEGs in CAV and CAD and plotted the scores in the subclustered UMAP. The CAV gene module mapped to the modulated SMC cluster, while the CAD gene module mapped to pericyte and fibroblast clusters (Fig. 3E). We next performed DGE analysis in modulated SMCs across disease conditions. In CAV, we identified *CXCL9* as the top unique DEG expressed by modulated SMCs, in addition to other IFN-induced genes (*CCL5, STAT1, HLA, IRF1* and *GBP4*) (**Supplemental Fig. 2A**).

To understand how modulated SMCs differ in CAV and CAD, we subclustered modulated SMCs and identified 4 unique subpopulations, with cell composition analysis showing enrichment of the IFN-signature (*CXCL9, IFI27*) subset in CAV and an enrichment of the *APOE* subset in CAD (**Supplemental 2B, C**). Pathway analysis revealed that modulated SMCs in CAV samples expressed genes related to allograft rejection, antigen presentation, and IFN signaling, while CAD modulated SMCs were enriched in AP-1 and ATF2 transcription factor networks and BDNF signaling pathways (**Supplemental 2D**). These findings indicate that modulated SMCs display striking plasticity across vascular disease etiologies where they differentially express IFN-activated genes and chemokines in CAV.

We next investigated transcriptional changes in fibroblasts. DGE analysis of fibroblasts revealed 1631 DEGs unique to CAV, 452 DEGs unique to CAD, and 805 shared DEGs (**Supplemental Fig. 2E**). Of the top DEGs unique to CAV, fibroblasts expressed *CXCL9, CST1, FAT1* and *POSTN*. Conversely, DEGs unique to CAD fibroblasts included *JUN, HSPA1A*, and *CHI3L2*. Subclustering of fibroblasts and cell composition analysis revealed that fibroblasts with a *POSTN* and *CCN2* signature were enriched in CAV relative to CAD and control samples while fibroblast subclusters in CAD showed expansion of an *APOE* expressing subset (**Supplemental Fig. 2F, G**).

In addition to SMC phenotype-switching, endothelial dysfunction is thought to be integral to CAV development at early stages following transplant^17^. DGE analysis of endothelial cells identified 1400 DEGs unique to CAV and 453 DEGs unique to CAD (**Supplemental Fig. 3A**). Top DEGs unique to CAV included *SMAD3*, and collagen genes (*COL4A1, COL4A2*). In CAD, upregulated DEGs included *SPP1* and *IER3*. We performed pathway analysis using the top 25 DEGs (**Supplemental Fig. 3B**). CAV endothelial cells were enriched in genes related to integrin expression, PDGFR signaling, and scavenger Class A receptors. CAD endothelial cells were noted to express genes enriched in pathways related to IFN signaling and responses to heme and amino acid deficiency. Subclustering of endothelial cells identified 5 subsets with distinct gene signatures (**Supplemental Fig. 3C, F**). Cell composition analysis revealed an expansion in a *PDE3A* endothelial subset in CAV (**Supplemental Fig. 3D**); Module scores for top endothelial DEGs genes in CAV and CAD were generated and plotted onto the endothelial cell UMAP, indicating that CAV genes mapped primarily to PDE3A endothelial cells, while CAD genes mapped primarily to *SELE* endothelial cells (**Supplemental Fig. 3E**).

Single-cell analysis revealed that modulated SMCs with IFN, fibroblast-like and proliferative gene signatures are enriched in CAV. These highly plastic, inflammatory cells resemble previously described phenotype-switched SMCs^25^. Endothelial cells and fibroblasts in CAV also show distinct profiles compared to CAD, and are defined by genes involved in adhesion, oxidative stress, IFN response, and vascular remodeling.

### CAV neointima is composed of modulated smooth muscle cells and myeloid cells

**Figure 4.**
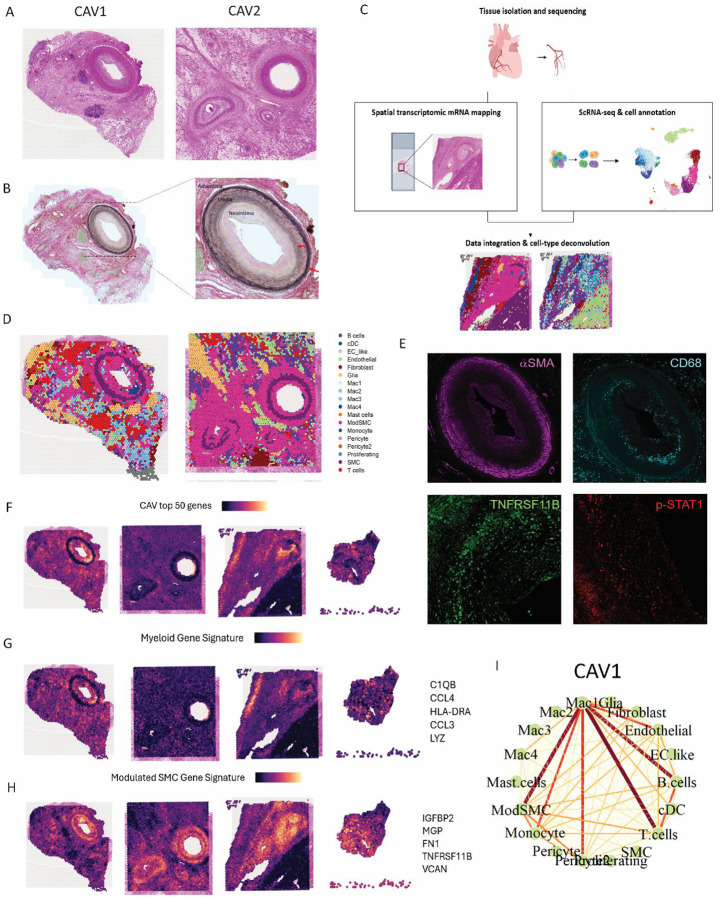
**A** Histopathology (H&E) of CAV1 and CAV2. **B** Verhoeff-Van Gieson (VVG) elastin staining of CAV1 and CAV2. **C** Graphic depicting single cell and spatial integration and deconvolution workflow. **D** Seurat deconvolution of CAV1 and CAV2 (first predicted cell state) using annotated single cell object containing high resolution myeloid and stromal cell states. **E** Immunofluorescence staining of select predicted cell types and genes (macrophages, CD68; smooth muscle cells, aSMA; modulated SMCs, TNFRSF11B; IFN signaling; Phospho-Stat1) to validate deconvolution results. **F** Spatial feature plot of the top 50 statistically significant DE genes identified from the CAV samples in the scRNA-seq dataset. **G** Spatial feature plot of a myeloid cell gene signature to validate spatial deconvolution results. **H** Spatial feature plot of a modulated SMC gene signature to validate spatial deconvolution results. **I** SPOTlight spatial interaction plot predicting which cells are likely to interact with each other based on proximity within a voxel in the CAV1 sample.

Histologically, CAV is defined by neointimal proliferation, which develops inward of the internal elastic lamina (Fig. 4A, B). Inward (or negative) vascular remodeling is a hallmark of the disease, yet the surrounding tissue (adventitia) provides additional pathological context. In some cases, the adventitial space in CAV may contain clusters of immune cells that form tertiary lymphoid organs (TLOs)—a phenomenon previously described in CAV^27^. We observed each of these features in coronary artery samples isolated from CAV patients in addition to dense cellular infiltrates within the adventitia (Fig. 4A, **Supplemental Fig. 4F**).

While histology provides valuable insights into structural changes, it does not reveal molecular drivers of disease. To address this gap, we performed spatial transcriptomics on four coronary arteries isolated from CAV explants. After preprocessing, QC and normalization we performed dimensionality reduction, clustering, and annotated spatial niches using marker genes (**Supplemental Fig. 4B,E**). we performed DimPlot visualizations to map annotated niches in space. In CAV samples 1 and 2 (CAV1 and CAV2), the neointimal niche was predominantly composed of SMCs, while the adventitial space contained primarily immune cells (**Supplemental Fig. 4C**). Cell composition analysis revealed expansion of immune cells and fibroblast clusters that varied across samples (**Supplemental Fig. 4D**), which may reflect differences in the capture area (i.e., proportion of the region of interest encompassing the adventitia versus the neointima).

To define spatially-restricted cell niches present within CAV, we integrated our scRNA-seq and spatial transcriptomics data (Fig. 4C–D, **Supplemental Fig. 4A**). The top DEG from CAV scRNA-seq samples spatially mapped most highly to the neointima (Fig. 4F). Using Seurat and SPOTlight deconvolution methods to identify where specific cell states map in space, we observed that the neointima was comprised of modulated SMC and myeloid subsets, including Mac1, Mac3, and Mac4 cells (Fig. 4D, **Supplemental Fig. 5A-F**). SMCs with a contractile gene signature (*ACTA2, MYH11, TPM2*) occupied the media (**Supplemental Fig. 5D**). The adventitia was occupied by an assortment of different cell types including B cells, T cells, and macrophage subsets (Mac3) as well as fibroblasts (Fig. 4D, **Supplemental Fig. 5F**). To strengthen deconvolution predictions, we plotted gene signature scores for contractile and modulated SMCs, myeloid, endothelial and fibroblast genes at the cell type and cell state levels onto the spatial data (Fig. 4F–H, **Supplemental Fig. 5D-F**). To validate these results at the protein level, we performed immunofluorescence staining of cell surface markers for myeloid, SMCs, modulated SMCs, endothelial cells, and type 1 IFN signaling (Phospho-Stat1) on the human CAV tissues utilized in spatial analysis (Fig. 4E, **Supplemental Fig. 6A-B,D,F**). Macrophages (CD68^+^), modulated SMCs (TNFRSF11B^+^) and type 1 IFN signaling gene Phospho-Stat1 were identified within the neointima (Fig. 4E, **Supplemental Fig. 6A**). SMCs (αMSA) were concentrated in the media and luminal neointima, with reduced expression in the deep neointima- a phenomenon previously described in coronary artery remodeling^28^. Consistent with prior studies, endothelial cells were identified in an intact layer on the most luminal aspect of the neointima^9^ (**Supplemental Fig. 6B**). Based on our DGE analysis, we also performed staining of Cathepsin S (CTSS), which was found to localize with myeloid (CD68+) cells in the neointima (**Supplemental Fig. 6F**). T cells have been suggested to serve as important primary mediators of CAV^29,30^. Spatial localization analysis and immunostaining indicated that they were not a dominant cell type in the neointima and instead were present within tertiary lymphoid organs (TLOs) found in the adventitial TLOs (Fig. 4A, D, **Supplemental Fig. 6C-E**). These T cell niches displayed transcriptional signatures consistent with T helper 1 and CD8 subsets (**Supplemental Fig. 6E**).

To identify cells which co-localize in space, Pearson correlation coefficients were calculated using SPOTlight. Spatial interaction analysis indicated that the strongest cell interactions in CAV samples were found between Mac1 macrophage and modulated SMCs and between Mac1 macrophages and T cells (Fig. 4I, **Supplemental Fig. 6G**) within the neointima. Together, these results highlight the prominent roles of macrophages and modulated SMCs within the neointima and suggest that these cell types may drive the inflammatory signals identified in our DGE analyses.

### Enrichment of IFN signaling in CAV

**Figure 5.**
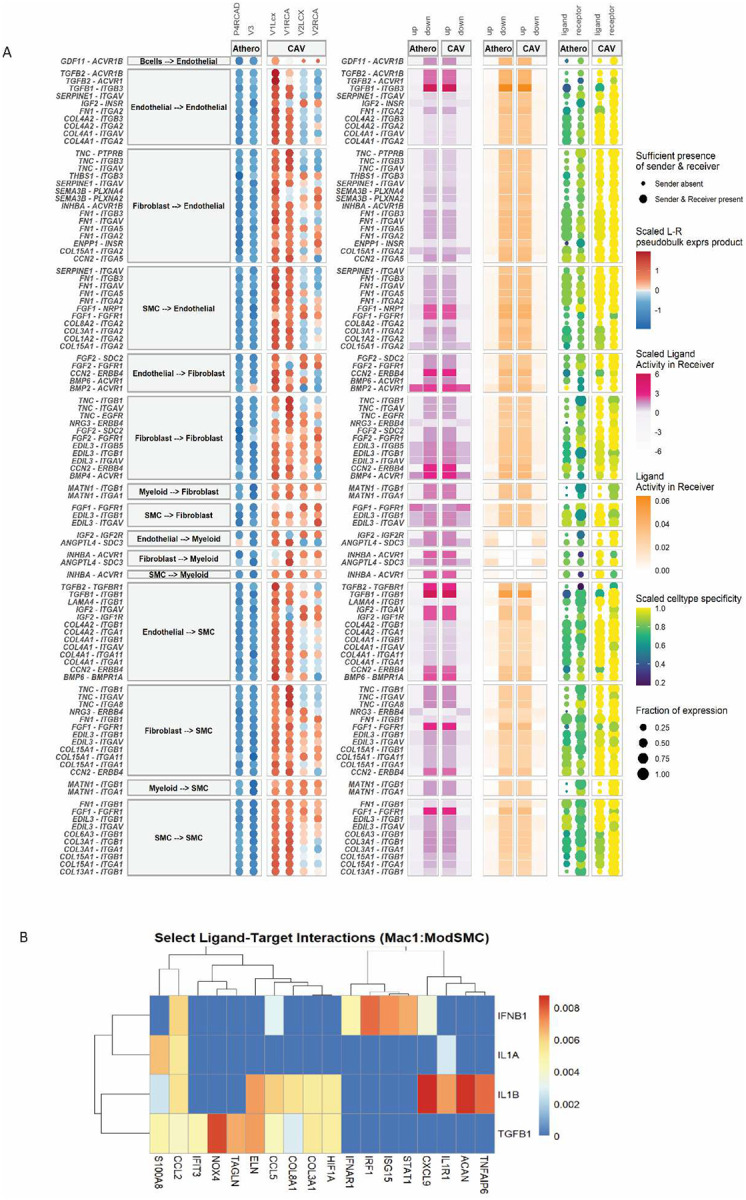
**A** MultiNicheNetr top 50 predicted ligand:receptor interactions in CAV samples. **B** Nichenet heatmap of top ligand:receptor interactions between Mac1 and modulated smooth muscle cells.

To identify specific ligand-receptor interactions in CAV, we leveraged *in silico* cell interaction analyses of the scRNA-seq dataset. MultiNicheNet analysis was performed on the global scRNA-seq object to compare the top ligand-receptor predictions in CAV and CAD samples. In CAV, top differentially expressed interactions were associated with *TGFβ* and cell adhesion signals (Fig. 5A). Integrins were among the top upregulated receptors, were primarily expressed in endothelial, fibroblast and SMCs, and were predicted to interact with a variety of extracellular matrix ligands including *TCN*, *EDIL3*, and various collagen genes in multiple cell types (Fig. 5A) (31). The top interaction predictions in CAD displayed minimal overlap with those in CAV, and included interactions between *LDLR* and *APOE, ICAM* and integrin interactions, and IL-10 signaling, which are all previously implicated in atherosclerosis pathogenesis or protection (**Supplemental Fig. 7**)^31–35^. These findings reinforce the notion that the top ligand-receptor interactions in CAV and CAD are distinct, highlighting fundamental differences in disease mechanisms.

To further dissect cell-specific contributions to CAV, NicheNet analysis was applied to individual sender and receiver cell types. Based on spatial interaction predictions, we focused on interactions between macrophages and modulated SMCs. Based on DEG analysis, spatial deconvolution, and immunoflourescence, we specifically explored interactions between Mac1 and modulated SMCs, as both clusters highly upregulated IFN signaling genes and localize to the neointima. The top Mac1 and modulated SMC interactions included type 1 IFN (*IFN*β*1*) signals from Mac1 inducing *IRF1*, *STAT1* and *ISG15* expression in modulated SMCs (Fig. 5b). Mac1 cells were also found to send other inflammatory signals to modulated SMCs including *IL-1*β and *TGF*β.

### Gene network analysis of CAV identifies a unique IFN expression module

**Figure 6.**
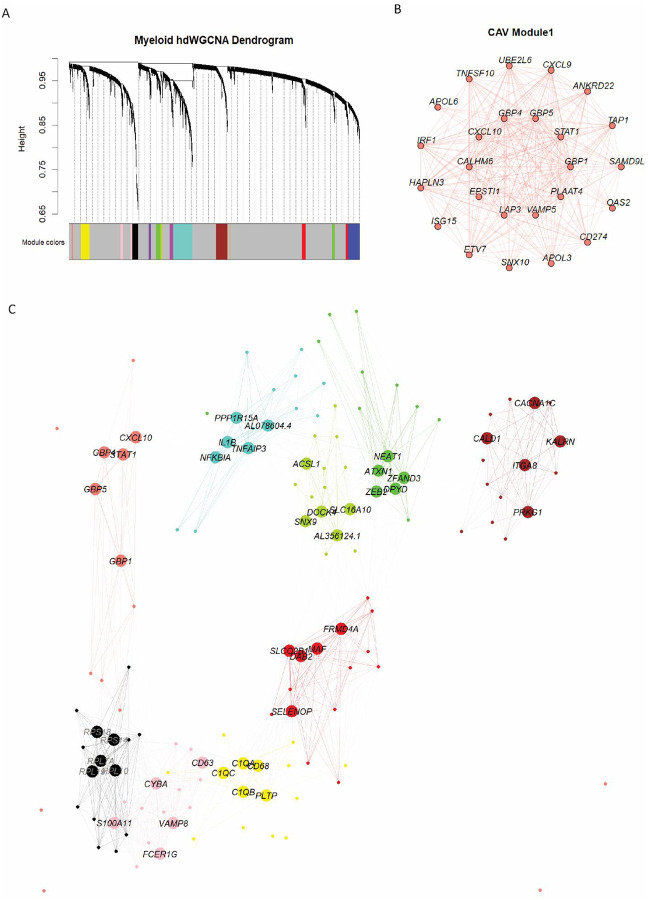
**A** Dendrogram depicting 13 unique gene expression modules identified from CAV scRNA-seq samples via Weighted Gene Correlation Network Analysis. **B** Module network plot depicting the top 25 genes within CAV Module 1. **C** Hub Gene Network Plot depicting the relationship of all 13 modules identified from CAV samples.

Given the complexity of cell signaling networks, we sought to identify co-expression networks specific to CAV. Using the CAV scRNA-seq dataset, we performed weighted correlation gene network analysis (WCGNA), identifying 13 unique functional expression modules (Fig. 6A). Similarly to our cell-cell communication analyses, IFN signaling represented a unique module in CAV samples (Fig. 6B). Interestingly, while both *IL1β* and IFN genes were identified as top ligand-receptor interactions in NicheNet analysis, WCGNA revealed that these expression modules are distinct (Fig. 6C). In conjunction with our NicheNet analysis, these findings highlight IFN signaling as a prominent and unique feature of CAV, independent of other inflammatory pathways and underscore its role as a distinct biological process within this disease state.

### Correlation of CAV to other type 1 interferonopathies

**Figure 7.**
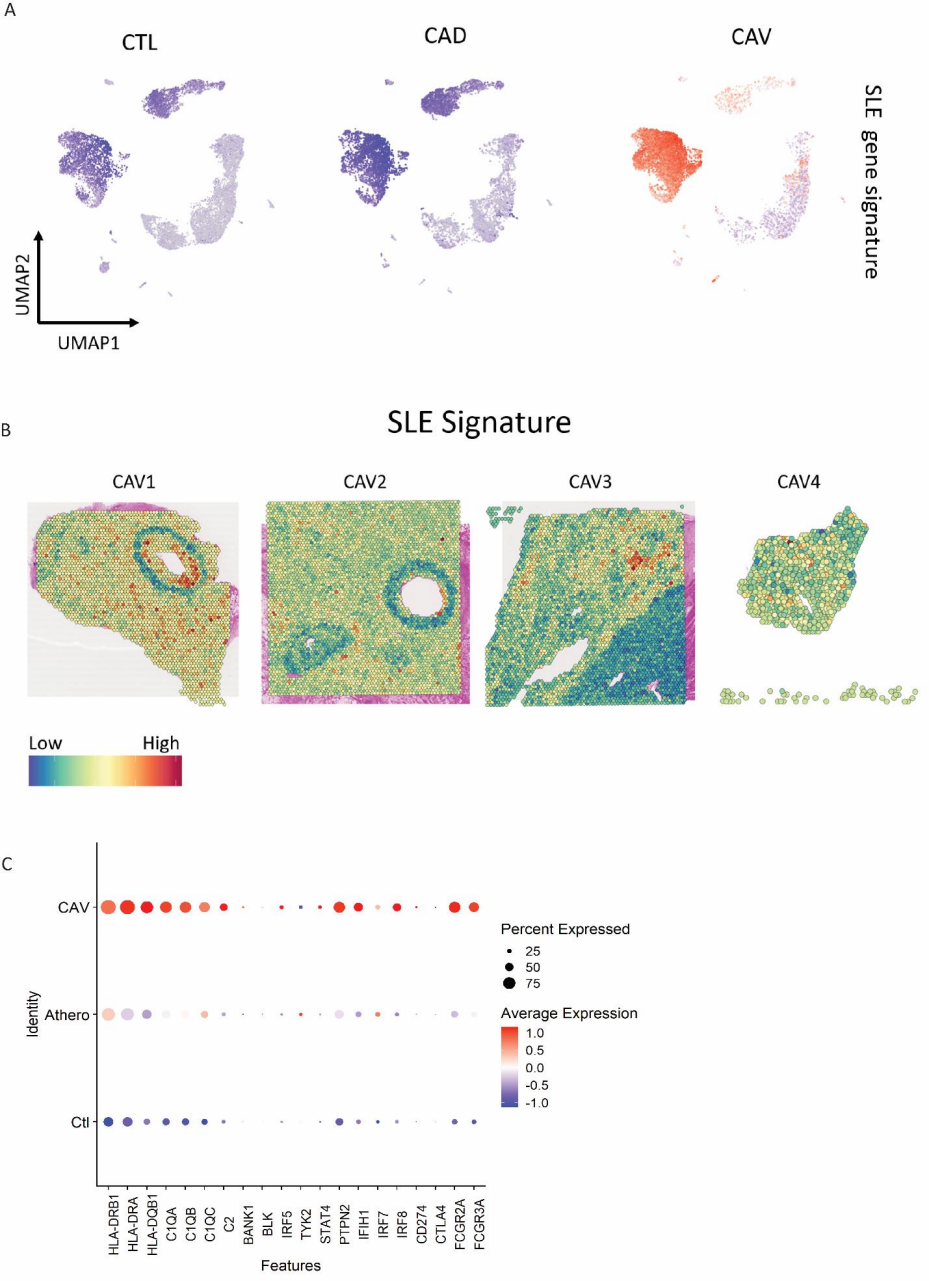
**A** Feature plot of a Systemic lupus erythmatosous (SLE) gene signature score **B** Spatial feature plot of SLE gene signature score within CAV spatial transcriptomic samples. **C** Dot plot of individual SLE gene expression in CAV, CAD and control samples scRNA-seq samples.

Given the prominence of IFN-related genes enriched in CAV samples, we investigated whether CAV shares broader similarities to IFN-mediated inflammatory diseases. We curated a list of canonical systemic lupus erythematosus (SLE) related transcripts including HLA genes (*HLA-DRB1, HLA-DRA, HLA-DQB1*), early complement genes (*C1QA, C1QB, C1QC, C2*), B cell signaling and development genes (*BANK1, BLK*), IFN signaling genes (*IRF5, TYK2, STAT4, PTPN2, IFIH1, IRF7, IRF8*), T cell regulatory genes (*CD274, CTLA4*) and immune complex clearance receptor genes (*FCGR2A, FCGR3A*) from a publicly available dataset^36^. Using this gene signature, we generated and plotted a signature score onto the scRNA-seq UMAP. SLE signature genes were highly and specifically expressed in CAV samples, particularly within myeloid cells, and showed minimal expression in CAD and control samples (Fig. 7A). We next investigated where these genes mapped in space and performed a spatial feature plot of the SLE gene signature. Interestingly, highest expression of the gene signature colocalized to voxels predicted to contain macrophage and modulated SMC populations within the neointima (Fig. 7B). To decipher which genes from the SLE signature were most highly expressed in CAV, we plotted the expression of individual SLE genes as a dot plot. This analysis revealed that HLA, complement, *PTPN2*, *IFIH1*, *IRF7*, *IRF8*, *FCGR2A* and *FCGR3A* genes were most associated with CAV (Fig. 7C). Collectively, our analysis suggests that CAV shares transcriptional similarities with another chronic, type 1 IFN-mediated inflammatory disease. These results also expand upon the notion that myeloid cells play an integral role in driving the disease, particularly regarding IFN signaling.

### Inhibition of interferon signaling pathways reduces incidence of CAV in mice

**Figure 8.**
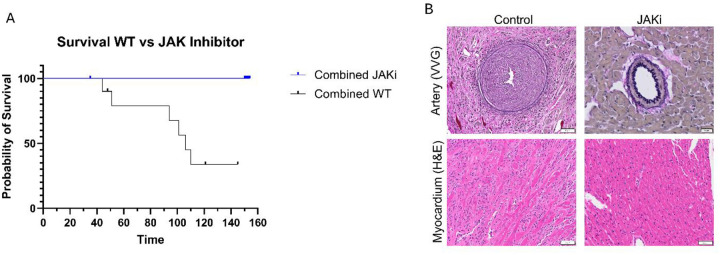
**A** Kaplan-Meier survival curve of control chow and Ruxolitinib (JAKi) chow treated allograft recipients (Mantel-Cox test, p=0.0034) **B** Histology (VVG and H&E) of arteries and myocardium of allografts collected from recipients that received control or JAKi chow. Control tissues were collected upon cessation of palpable heartbeat. JAKi tissues were collected at 150 days post-transplant.

To address whether type 1 IFN signaling pathways influence transplantation outcomes in CAV, we assessed the efficacy of Ruxolitinib, a JAK1/JAK2 inhibitor, in a BM12 mouse model of CAV. Compared to controls, recipients treated with Ruxolitinib had significantly improved allograft survival over a 150-day period (Fig. 8A). We assessed the severity of vasculopathy by Verheoff-van Gieson (VVG) staining and demonstrate that Ruxolitinib-treated recipients had drastically reduced incidence of CAV compared to controls (Fig. 8B). Lastly, BM12 allografts from recipients treated with Ruxolitinib also showed improved histology at 150 days post-transplant, compared to controls harvested upon cessation of a palpable heartbeat (Fig. 8B). These results corroborate our transcriptomic analysis and suggest that inhibition of type 1 IFN signaling can improve transplantation outcomes by reducing the incidence of CAV.

## Discussion

This study utilized scRNA-seq and spatial transcriptomics of human coronary arteries to provide a detailed, high-resolution characterization of the cellular and molecular signals driving CAV pathogenesis. To our knowledge, this is the first study to apply this integrative approach to CAV. While a prior study employed spatial technologies to study CAV, it lacked high resolution due to the utilized spatial technology, and the study controls were limited to arteries with less advanced CAV progression. By comparing distinct cardiovascular diseases (CAV vs CAD), we identified gene signatures unique to CAV and comprehensively defined spatial niches in diseased samples. Importantly, we highlighted key molecular distinctions between CAV and CAD. Our findings revealed several potential therapeutic targets for *in vivo* studies. Additionally, we expanded on the role of IFN signaling in CAV. Historically thought to be dominated by type 2 IFN signaling from activated T cells, our data instead suggest that myeloid cells play a central role as intermediaries in sustaining a chronic inflammatory state via type 1 IFN sensing and production. We demonstrate in a mouse model of CAV that blockade of JAK1 and JAK2 signaling with Ruxolitinib reduces vasculopathy and prolongs allograft survival. These insights provide a deeper understanding of CAV pathology and open avenues for the development of CAV-specific therapeutic strategies.

Endothelial cells are widely thought to initiate CAV pathogenesis, due to their immediate exposure to ischemia reperfusion injury (IRI) and shear stress^37,38^. Their dysfunction is a known predictor of disease progression and promotes T cell recruitment, donor antigen presentation, and cytokine release that propagates downstream inflammation^9,17,37,38^. Interestingly, in our dataset, endothelial cells did not highly express HLA markers, but did show upregulation of integrin genes, inflammatory cytokines, IFN-stimulated genes and markers of dysfunction. DGE analysis identified several CAV-specific genes in endothelial cells, including *SMAD3* and *IFI27*, an IFNα sensing gene that has also been cited as a marker of EndoMT^9,39,40^. Other top upregulated genes include NEAT1 and PDE4B, which have previously been implicated in endothelial proliferation and cardiac injury responses, respectively^41,42^. In cell-cell interaction analyses, endothelial cells were predicted to signal to macrophage subsets via IL-15, IL-6 and IL-33, which may contribute to macrophage activation. Together, our findings are consistent with prior studies implicating endothelial cells in CAV pathogenesis^25,43^.

Intimal thickening is the defining pathological feature of CAV, directly contributing to vascular occlusion, tissue hypoxia, and allograft failure. This phenomenon is largely driven by SMC proliferation and extracellular matrix production^43^. In our data, there is a clear distinction between medial SMCs, which retain a contractile gene signature, and neointimal SMCs which adopt a modulated gene signature. These modulated SMCs lose contractile markers and upregulate genes associated with fibroblast-like, inflammatory, and proliferative states^9,44^, including IGFBP2, TNFRSF11B, FN1, BGN, THBS2, COL8A1 and CCN2.

Phenotype switching in SMCs is thought to result from sterile injury and cytokine exposure^45–47^. Our signaling analysis suggests that it is driven by both immune and stromal cues. Macrophages appear to play a central role, secreting IL-1β, TNF, TGFβ, PDGF, and IGF-1, which are factors thought to induce SMC proliferation, fibrosis and inflammation^48–52^. Notably, depleting macrophages in preclinical models of CAV mitigates neointimal thickening, reinforcing their pathogenic role^23^. Stromal-derived signals, including TGFβ, CCN2 and TGM2 also target SMCs, promoting fibrotic remodeling and cell migration^50,53,54^. In our data, modulated SMCs expressed chemokines (*CXCL9, CCL19, CCL5, CXCL10*) and integrins such as CD44, suggesting that they may actively recruit and adhere immune cells^55–57^. This reciprocal signaling between stromal and immune compartments likely sustains chronic inflammation and drives CAV progression, offering a potential therapeutic target.

CAV is widely regarded as an immune-mediated disease, yet research has predominantly focused on the role of T cells and donor-specific antibodies^18–22^. In our data, T cells demonstrated activated gene signatures and were observed to accumulate in organized TLOs in some samples^27^. However, one surprising observation from our spatial data was the rare presence of T cells within the neointima, a finding that contrasts with previous studies emphasizing infiltrating T cells as drivers of CAV pathogenesis. While alloreactive T cells are a widely appreciated mechanism of acute allograft rejection, we challenge the notion that T cells are primary mediators of CAV. There is a growing appreciation of the diverse roles of macrophages in both allograft tolerance and rejection; however, few studies have investigated the role of myeloid cells in CAV.

At a global level, myeloid cells were significantly enriched in CAV samples, and DGE were largely driven by these cells. Notably, DEGs included IFN-stimulated genes and *CTSS* (Cathepsin S), a potent elastase exclusively expressed in myeloid cells. Previous studies on *CTSS* suggest its involvement in elastin disorganization and breakdown following myeloid recruitment, leading to intimal hyperplasia^58^. Via immunofluorescence, we found that CTSS localized to CD68+ cells within the neointima of CAV samples. This suggests a potential mechanism by which myeloid cells facilitate medial SMC migration into the lumen. Further subclustering of myeloid cells revealed a significant expansion of the Mac1 subset, characterized by a strong type 1 IFN signature. Spatial analysis demonstrated that this subset localized to diseased niches and was predicted to interact with modulated SMCs and T cells—two cell types known to contribute to CAV pathogenesis. Cell signaling analysis revealed that Mac1 macrophages signal to modulated SMCs via type 1 IFN (IFNβ1), a process we hypothesize is critical for both SMC phenotype switching and the maintenance of inflammation within the neointima.

The clinical features of CAV share striking similarities with those observed in chronic autoimmune diseases, particularly interferonopathies. For example, Systemic Sclerosis (SSc), a chronic interferonopathy, is also characterized by vasculopathy that is driven by type 1 IFN signaling^59,60^. Preclinical studies of other autoimmune diseases have also demonstrated that type 1 IFN plays a key role in the development of vasculopathy^61^. Furthermore, downstream genes induced by type 1 IFN signaling, such as *ISG15*, have been implicated in endothelial dysfunction^62^, a mechanism involved in the initiation of CAV. DGE and cell signaling analyses of our single cell data highlight the prominent role of type 1 IFN in CAV. Type 1 IFN genes were upregulated across nearly all cell types in CAV samples, and were unique to CAV compared to CAD and control samples. Traditionally, CAV research has focused on the role of type 2 IFN secretion by T and NK cells in neointimal development^63,64^. We propose that type 1 IFN signaling is central to maintenance of chronic inflammation in CAV, and that macrophages are key mediators of this phenomenon. Macrophages classically act as intermediaries in type 1 IFN signaling due to their high expression of pattern recognition receptors (PRRs), which detect and respond to stimuli that trigger IFN production. These cells are also highly sensitive to IFN signaling, creating a self-amplifying feedback loop that sustains inflammation.

Notably, other chronic type 1 interferonopathies are often triggered and maintained by PRR sensing of free nucleic acids or immune complexes. In the context of CAV, this process may be perpetuated by donor-specific antibodies or the release of donor antigens from apoptotic cells, which induce PRR sensing^65,66^. These inflammatory cascades perpetuate themselves by inducing further apoptosis, thereby accelerating disease progression. To further explore the role of type 1 IFN in CAV, we compared SLE gene signatures to our single cell data. SLE-associated genes were highly expressed in CAV samples, particularly in myeloid cells. The role of myeloid sensing and amplification of type 1 IFN signaling may underlie key pathological effects, including endothelial dysfunction and vasculopathy, providing new insights into the mechanisms of CAV progression and potential therapeutic targets. Drawing parallels between CAV and interferonopathies is useful, as the latter have established biomarkers and effective IFN-targeted therapies, such as JAK/STAT inhibitors^67^. Expanding on this idea, we tested the efficacy of type 1 IFN inhibition in a mouse model of CAV and demonstrated that inhibition of IFN signaling drastically reduced the incidence of vasculopathy in treated recipients and significantly prolonged allograft survival. These studies highlight the potential benefit of type 1 IFN inhibition in CAV, opening the door for future studies to further characterize this therapeutic strategy.

Our study is not without limitations. Given the involvement of some level of atherosclerosis in most adult coronary arteries, the control samples in our dataset are not perfectly healthy tissue. However, the utilized controls are CAV-free, so we were still able to appropriately compare CAV to a distinct coronary artery disease. Additionally, there is scarce availability of FFPE coronary arteries with CAV that have acceptable RNA quality for spatial transcriptomics, limiting our analysis to a small sample size. Furthermore, our spatial analysis was limited only to CAV samples, and the mRNA quality is substandard than that of scRNA-seq data, which necessitates the integration of spatial data with higher resolution datasets to provide appropriate transcriptional context. Using single-cell data to deconvolute spatial slices allows for higher-resolution analysis, but disparities between the datasets can pose limitations. In single-cell samples, cardiomyocytes are underrepresented. This scarcity may result in biased voxel imputations, where spatial voxels that likely correspond to cardiomyocytes are misclassified as other cell types. This discrepancy could explain why modulated SMCs are predicted to occupy the adventitial space around arteries, despite prior literature indicating these cells are typically confined to the neointima^68^. Additionally, the CAV samples used in the spatial analysis are examples of advanced CAV, so we do not have a means to spatially profile CAV at early stages.

In conclusion, CAV is a complex disease driven by chronic inflammation, immune activation, and SMC phenotype-switching to a proliferative, inflammatory state. Using high resolution single-cell and spatial transcriptomics, we identified macrophages as key mediators of CAV progression which sustain neointimal inflammation through type 1 IFN. Our findings highlight the interplay between macrophages and SMCs while uncovering a previously unappreciated role for type 1 IFN signaling in disease progression. These results provide novel insights into CAV pathogenesis and lay the groundwork for future *in vivo* studies to validate potential therapeutic targets.

## Supplementary Material

Supplementary Files

This is a list of supplementary files associated with this preprint. Click to download.

- CAVmanuscriptsupplementalfigures10825.pdf

## Nonstandard Abbreviations and Acronyms

CAV: Cardiac allograft vasculopathy
CAD: Coronary artery disease
CTL: Control
IVUS: Intravascular ultrasound
mTOR: Mammalian target of rapamycin
SMC: smooth muscle cells
IFN: Interferon
scRNAseq: Single-Cell RNA Sequencing
UMAP: uniform manifold approximation and projection
DGE: differential gene expression
DE: differentially expressed
TLO: tertiary lymphoid organ
SLE: Systemic Lupus Erythematosus
